# Hypoxia-Inducible Factor-1: A Potential Target to Treat Acute Lung Injury

**DOI:** 10.1155/2020/8871476

**Published:** 2020-11-17

**Authors:** Yang Liu, Du Xiang, Hengcheng Zhang, Hanlin Yao, Yanfeng Wang

**Affiliations:** ^1^Zhongnan Hospital of Wuhan University, Institute of Hepatobiliary Diseases of Wuhan University, Transplant Center of Wuhan University, Wuhan 430071, China; ^2^Transplantation Research Center, Renal Division, Brigham and Women's Hospital, Harvard Medical School, Boston, 02115 MA, USA

## Abstract

Acute lung injury (ALI) is an acute hypoxic respiratory insufficiency caused by various intra- and extrapulmonary injury factors. Presently, excessive inflammation in the lung and the apoptosis of alveolar epithelial cells are considered to be the key factors in the pathogenesis of ALI. Hypoxia-inducible factor-1 (HIF-1) is an oxygen-dependent conversion activator that is closely related to the activity of reactive oxygen species (ROS). HIF-1 has been shown to play an important role in ALI and can be used as a potential therapeutic target for ALI. This manuscript will introduce the progress of HIF-1 in ALI and explore the feasibility of applying inhibitors of HIF-1 to ALI, which brings hope for the treatment of ALI.

## 1. Introduction

Acute lung injury (ALI) refers to increased alveolar-capillary permeability caused by severe noncardiogenic factors such as severe infection, shock, and burns, resulting in diffuse pulmonary interstitial and parenchymal edema [1]. The clinical manifestations of ALI are progressive hypoxemia or acute respiratory distress syndrome (ARDS) and can even progress to fatal respiratory failure [2, 3]. ALI/ARDS is a disease that endangers human health worldwide. Although research on the pathogenesis and molecular mechanism of ALI/ARDS has made remarkable progress, its morbidity and fatality rate are still high. Recent studies have reported that the incidence of ALI/ARDS is 1%~4%, the case fatality rate is 22% ~65%, and the case fatality rate in the pediatric population is approximately 24%; thus, it is urgent to find an effective treatment for ALI [4]. In the pathogenesis of ALI/ARDS, various factors stimulate the production and accumulation of a large number of inflammatory cells (neutrophils, mononuclear macrophages, etc.) in the lung. At the same time, inflammatory cells activate and release proinflammatory factors such as reactive oxygen species (ROS), tumor necrosis factor *α* (TNF-*α*), interleukins (ILs), and elastase, which promote the deterioration of the inflammatory response [5, 6]. ROS can destroy pulmonary microvascular endothelial cells and epithelial cells in different ways, increase pulmonary vascular permeability, and lead to the formation of pulmonary edema. In addition, ROS can interact with inflammatory factors and promote the expression of inflammatory factor-related proteins, resulting in an inflammatory cascade [7, 8]. Hypoxia-inducible factor-1 (HIF-1) is an oxygen-dependent transcriptional activator that was first discovered in 1992 [9]. HIF-1 is widely expressed in tissue during hypoxia, and it plays an important pathophysiological role in maintaining oxygen homeostasis in the body by regulating the expression of a series of genes related to the adaptation to hypoxia [9]. HIF-1 is also an important inflammatory regulator involved in the regulation of acute and chronic inflammation [10, 11]. Interestingly, under hypoxic conditions, ROS released from the mitochondrial electron transport chain can participate in the regulation of HIF-1 activity [12]. Previous studies have reported that HIF-1 plays an important role in ALI and participates in inflammation, apoptosis, and pulmonary fibrosis by regulating corresponding target genes, but its specific mechanism is not very clear. Our review focuses on the interaction between HIF-1 and ROS and the role of HIF-1 in the pathogenesis of ALI and the progress in the treatment of ALI with HIF-1 inhibitors.

## 2. HIF-1

### 2.1. The Structure of HIF-1

**Figure 1:**
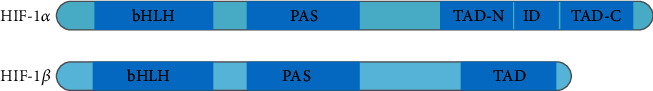
The structures of HIF-1 *α* and HIF-1 *β*. bHLH: helix-loop-helix; PAS: Per-aryl hydrocarbon receptor nuclear translocator-Sim; TAD: transcription activation domain; ID: inhibitory domain.

HIF-1 is a heterodimer composed of the functional subunit HIF-1*α* (120 kD) and the structural subunit HIF-1*β* (91~94 kD) [13]. The human HIF-1*α* gene is located on chromosome 14 (14 q21-24) and has a length of 3720 bp, and the protein it encodes is an 826-amino acid polypeptide. The length of the human HIF-1*β* gene is 2604 bp, and the protein it encodes is a 774- or 789-amino acid polypeptide [13]. HIF-1*α*, a regulatory and active subunit, is a unique subunit structure of HIF-1. HIF-1*β*, also known as aryl hydrocarbon receptor nuclear translocator (ARNT), is a subunit of an aromatic hydrocarbon receptor complex that can form dimers with proteins. The *α* and *β* subunits have the following structure: (1) the amino-terminal half of each subunit contains a basic helix-loop-helix (bHLH) that defines a large superfamily of dimeric eukaryotic transcription factors in which the HLH domain mediates dimerization and the basic domain contacts DNA; (2) the Per-aryl hydrocarbon receptor nuclear translocator-Sim (Per-ARNT-Sim; PAS) domain contributes to the formation of protein dimers; and (3) the carboxyl terminus of each subunit contains a transcription activation domain (TAD), which can bind to the transcription initiation complex to regulate gene transcription [13] (Figure 1). TAD in the HIF-1*α* structure contains TAD-N and TAD-C, which can participate in transcriptional activation. There is an inhibitory domain (ID) between TAD-N and TAD-C, which can significantly reduce the activity of TAD under normoxia.

### 2.2. The Regulation in HIF-1 Expression

**Figure 2:**
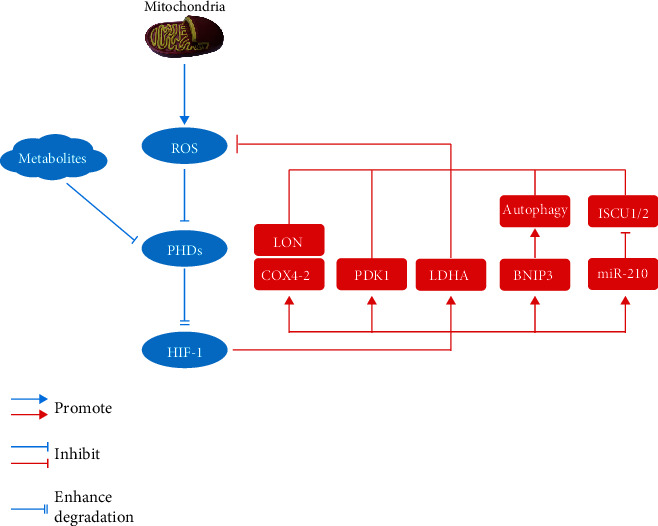
The interplay between HIF-1 and ROS under hypoxic condition. PHD: prolyl hydroxylase; LON: mitochondrial ATP dependent matrix protease; COX-4: cytochrome c oxidase IV-2; PDK-1: pyruvate dehydrogenase (PDH) kinase 1; LDHA: lactate dehydrogenase A; BNIP3: BCL2 and adenovirus E1B 19 kDa-interacting protein 3; ISCU1/2: iron–sulfur cluster assembly protein.

The *α* subunit is a unique regulatory and active subunit of HIF-1 [14]. The stability and transcriptional activity of the HIF-1 protein are both regulated by intracellular oxygen concentration [15, 16]. Therefore, the physiological activity of HIF-1 mainly depends on the activity and expression of the HIF-1*α* subunit [16]. The *β* subunit is shared by many transcription factors, and it can be constitutively expressed in the nucleus without being regulated by the oxygen concentration. The function of the *β* subunit may be related to maintaining the structural stability of HIF-1 and the active conformational changes caused by dimerization [17]. HIF-1*α* must form a heterodimer with HIF-1*β* to become active HIF-1 [17]. HIF-1 is a core transcription factor that induces hypoxia genes and repairs the internal microenvironment of cells. HIF-1*α* is an oxygen regulatory protein that is very sensitive to the concentration of oxygen and is called the “master switch of hypoxia gene expression” [18]. Under normal oxygen conditions, the oxygen-dependent degradation domain (ODDD) on the HIF-1 gene is hydroxylated by prolyl hydroxylase (PHD), binds with ubiquitin E3 ligase (von Hippel-Lindau, pVHL), and is ultimately degraded through the protein ubiquitination hydrolysis system [19, 20]. When the cell is in a hypoxic state or the function of pVHL is lost, the mitochondrial electron transport chain is also inhibited. In addition, the accumulation of ROS and various metabolites such as lactic acid and pyruvate also inhibits PHD and reduces the degradation of HIF-1 and stabilizes its expression [21, 22] (Figure 2). HIF-1*α* accumulates and is transported to the nucleus to forming the HIF-1 complex following binding with HIF-1*β* [23]. In addition, HIF-1*α* acts as a transcription factor in conjunction with the hypoxia reaction element (HRE) to further activate the transcription of multiple downstream target genes, such as vascular endothelial cell growth factor (VEGF), erythropoietin (EPO), and induced nitric oxide synthase (iNOS), which in turn triggers a series of oxygen-resistant adaptive responses in tissues and cells [24].

### 2.3. The Downstream Target Genes and Functions of HIF-1

The target genes regulated by HIF-1 all contain HREs. After HIF-1*α* specifically recognizes and binds to the core HRE sequence (5′-RCGTG-3′), it exerts a transcriptional activation effect and activates the transcription of target genes [25]. These activated genes are as follows: (1) angiogenesis-related genes such as VEGF and its receptor [26]; (2) various enzymes involved in glucose metabolism, energy metabolism, and cell proliferation such as adenosine kinase (AK), pyruvate kinase M2 (PKM2), phosphoglycerate kinase 1 (PGK1), hexokinase II, pyruvate dehydrogenase kinase (PDK), lactate dehydrogenase (LDH), glucose transporters (GLUT), glyceraldehyde-3-phosphate dehydrogenase (GAPDH), and transforming growth factor (TGF-*β*) [27–31]; (3) red blood cell metabolism- and iron metabolism-related genes such as erythropoietin (EPO), transferrin, and transferrin receptor-1 (TfR1) [32, 33]; and (4) heme oxygenase (HO) and iNOS [34, 35]. HIF-1*α* regulates angiogenesis, energy metabolism, red blood cell production, cell proliferation and survival, vascular remodeling, and the vasomotor response by regulating the expression of its downstream target genes so that hypoxic tissues and cells can maintain oxygen homeostasis to tolerate hypoxia [26–30, 36–38].

## 3. HIF-1 and ROS

### 3.1. ROS

ROS are unstable compounds containing highly reactive oxygen molecules that are produced by redox reactions [39]. ROS include oxygen-containing free radicals (superoxide anion free radicals, hydroxyl free radicals, etc.), nonradical derivatives of oxygen, hydroperoxides, and lipid peroxides [40–43]. Among them, oxygen-free radicals mainly refer to superoxide anion radicals (O2•-), hydroxyl radicals (•OH), peroxides (RO_2_), and oxygen organic-free radicals (RO•) [44–46]. ROS are produced and exert their biological effects in mitochondria. ROS produced by mitochondria can be released into the cytoplasm through specific mitochondrial ion channels/transporters, such as the mitochondrial permeability transition pore (mPTP) [47]. ROS can also be generated by extramitochondrial enzymes such as reduced nicotinamide adenine dinucleotide phosphate oxidase (NOX), lipoxygenase, xanthine oxidase, myeloperoxidase, and cytochrome P450 cyclooxygenase [48–51]. There is a complex antioxidant system in the body that can produce and remove ROS in a dynamic balance under normal circumstances to avoid causing damage to the body [52]. In some specific cases, the accumulation of ROS in cells increases, which can cause excessive oxidation of proteins, lipids and, DNA, resulting in varying degrees of cell damage [53]. ROS bind to proteins to produce carbonyl derivatives, which change the tertiary structure of proteins, resulting in partial or full unfolding, and easily form protein-protein crosslinks, resulting in protein denaturation or the loss of enzymatic activity [54]. The main physiological functions of ROS in the body include participation in the immune response and biological aging process, as well as the regulation of some signaling pathways [55, 56].

Excessive production of ROS is key to the development of ALI, which can affect the activation of lung inflammatory cells and the release of inflammatory mediators and can also damage the alveolar-vascular endothelial cell barrier structure, resulting in a decrease in barrier stability [57]. Under physiological conditions, the elimination of ROS depends on the effectiveness of antioxidant substances in the body to maintain a dynamic balance. However, under pathological conditions, such as acute inflammation, the amount of ROS produced far exceeds the scavenging capacity of the antioxidant system. Excessive ROS accumulate in the body and destroy the structure and function of tissues and organs and cause metabolic disorders through oxidative stress and inflammation cascade amplification [58].

### 3.2. The Impact of ROS on ALI

ROS oxidize or damage proteins, lipids, and DNA, thereby directly or indirectly inducing protein denaturation and lipid peroxidation. If the excessive accumulation of ROS exceeds the elimination ability of the body's antioxidant system, cell and tissue damage occur. The oxidative effect of ROS mediates lung injury [59, 60] through the following mechanisms: (1) ROS oxidize phospholipids on the surface of cell membranes, causing fatty acid chain breaks, membrane structure disorders, and decreased fluidity and permeability, resulting in lung dysfunction. (2) ROS oxidize lipid molecules to produce a large number of vasoactive substances and proinflammatory molecules, such as promoting the formation of a large number of thromboxanes, thereby increasing the degree of pulmonary vascular tension and inflammation. (3) ROS oxidize the amino acids in the protein-peptide chain, destroy the basic molecular structure of the protein, and change the activity and function of the protein. ROS antioxidant enzymes cause oxidation and antioxidation imbalance in the body. (4) ROS activate transcription factors through oxidation, such as nuclear factor kappa-B (NF-*κ*B) and protease activating factor-1 (Apaf-1), to upregulate inflammation-targeting proteins (intercellular adhesion molecules, cyclooxygenase-2, etc.), thereby promoting inflammation [61]. (5) ROS can oxidize DNA and cause DNA base deletions, point mutations and strand breaks, causing cell damage and even cell death. Increased ROS production can destroy the microstructure of cells through self-oxidation, which can cause structural or functional damage to pulmonary vascular endothelial cells, epithelial cells, and smooth muscle cells, resulting in dysfunction of the alveolar-vascular endothelial barrier and vasomotor function, thereby promoting the progression of ALI.

### 3.3. The Interaction of ROS and HIF-1

Mitochondria are the main organelles associated with cellular oxygen consumption and the main site of ROS production. HIF-1 is a transcription factor that regulates hypoxia adaptation. Under hypoxic condition, ROS are involved in the regulation of HIF-1 activity. A hypoxic environment promotes the production of ROS in mitochondria, and a large amount of ROS can promote the stability of the HIF-1 protein under hypoxic conditions [62]. In addition, hypoxia may initially promote oxidative stress through mitochondrial ROS production. The increased ROS can upregulate HIF-1, and activated HIF-1*α* can cause persistently high levels of oxidative stress by promoting the production of oxidative products or inhibiting the antioxidant effect [63–66]. Interestingly, HIF-1 also has the ability to inhibit oxidative phosphorylation and ROS generation [67], which indicates that a negative feedback loop in which ROS-mediated regulation of HIF-1 plays a key role. HIF-1 can prevent the excessive production of mitochondrial ROS under hypoxic conditions rather than promote oxidative stress. Studies have shown that the expression of HIF-1 in cells in vivo and in vitro can prevent excessive accumulation of ROS and inhibit cell apoptosis [68–71]. Therefore, maintaining a balance between HIF-1 and ROS is essential. The possible mechanism by which HIF-1 reduces ROS generation under hypoxia [71] is as follows: (1) Cells express the COX4-1 (cytochrome c oxidase IV-1) regulatory subunit of cytochrome c oxidase (ETC complex IV) under aerobic conditions but switch to the COX4-2 (cytochrome c oxidase IV-2) subunit under hypoxic conditions. HIF-1 activates transcription of the genes encoding COX4-2 and LON (a mitochondrial protease that is required for the degradation of COX4-1). Under conditions of chronic hypoxia, ETC complex IV can also become a source of increased ROS production if the COX4 subunit switch does not occur [72]. (2) HIF-1 activates the gene encoding pyruvate dehydrogenase (PDH) kinase 1 (PDK1), which shunts pyruvate away from the mitochondria [68]. PDK1 and LDHA (lactate dehydrogenase A) cooperate to decrease ETC flux and offset the reduced f electron transport efficiency under hypoxic conditions to reduce the production of ROS [73]. (3) HIF-1 activates the gene encoding BNIP3 (BCL2 and adenovirus E1B 19 kDa-interacting protein 3), which triggers selective mitochondrial autophagy [74]. (4) MicroRNA-210 is induced, which blocks iron-sulfur cluster assembly proteins (ISCU1/2) that are required for oxidative phosphorylation [75] (Figure 2).

## 4. HIF-1 and Inflammation

The inflammatory response requires the interaction of a series of cytokines, and HIF-1*α* is an important factor associated with gene regulation during the inflammatory response. There is a positive feedback proinflammatory effect in ALI, and a large number of inflammatory factors upregulate HIF-1. Increased HIF-1 stimulates the release of inflammatory factors, amplifies the inflammatory response, and exacerbates lung injury [76]. Li et al. [77] showed that intracellular succinate induced angiogenesis through HIF-1*α* induction, which is related to the activation of the VEGF gene. HIF-1*α* accumulates under hypoxic conditions and can bind with the VEGF gene promoter to induce VEGF expression [77]. In allergic airway diseases, VEGF is an effective stimulator of inflammation and can promote airway remodeling and physiological dysfunction [78, 79]. In a mouse model of allergic airway disease caused by the inhalation of ovalbumin (OVA), the expression levels of HIF-1*α* and VEGF increased, and the production of inflammatory mediators, such as IL-4, IL-5, and IL-13, significantly increased, airway hyperresponsiveness and pulmonary vascular permeability increased, which confirmed that the inhibition of HIF-1*α* could reduce antigen-induced airway inflammation and hyperresponsiveness by regulating VEGF-mediated vascular leakage [80]. Under normoxic conditions, interleukin-1*β* (IL-1*β*), IL-6, TNF-*α*, and other cytokines are upregulated by HIF-1. Under hypoxia, cytokines can increase HIF-1 activity [81].

The NF-*κ*B family controls multiple processes, including immunity, inflammation, tumor cell proliferation, and nervous system function [82]. Mo et al. [83] showed that the expression of NF-*κ*B in alveolar of ALI/ARDS patients was abnormally increased, and the activation of NF-*κ*B may contribute to the increased expression of a variety of cytokines in the lung. Chen et al. [84] confirmed that NF-*κ*B was abnormally upregulated in the alveolar cells of ALI patients and regulated the production of a large number of gene products with NF-*κ*B sites, such as IL-1*β*, IL-6, and TNF-*α*. The interaction of various cytokines and inflammatory factors initiates the inflammatory cascade, which ultimately leads to ALI [85, 86]. In addition, HIF-1*α* can act on various adenylate receptors, thereby alleviating lung injury [87, 88]. For example, HIF-1*α* can effectively reduce the severity of ALI and prolong patient survival by regulating A2BAR (adenosine receptor A2b) [89].

## 5. HIF-1 and Apoptosis of Alveolar Epithelial Cells

Alveolar type II (AT-II) cells are also known as granular alveolar cells. AT-II cell apoptosis occurs in the early stage of ALI, leads to the loss of a large number of alveolar epithelial cells, and ultimately promotes the progression of ARDS [90]. In the progression of ALI, HIF-1*α* can increase glucose metabolism in tracheal epithelial cells, thereby increasing lung ventilation and improving pulmonary edema and respiratory distress. However, more studies have confirmed that HIF-1*α* promotes AT-II cell apoptosis. This effect may be related to different signaling pathways regulated by HIF-1*α*. Krick et al. [91] found that hypoxia suppressed alveolar epithelial cell proliferation and enhanced AT-II cell apoptosis through activation of the HIF-1*α*/HRE axis and a mechanism that involves Bnip3L; thus, targeting HIF-1*α* may represent a new strategy to attenuate the degree of acute lung injury. He et al. [92] suggested that hypoxia-induced apoptosis in AT-II cells could be alleviated by inhibiting the HIF-1*α*-HRE axis.

## 6. HIF-1 and Pulmonary Fibrosis

Pulmonary fibrosis (PF) after ALI is a process in which lung inflammation leads to persistent alveolar damage, the production of extracellular matrix, repeated cell destruction, repair, and reconstruction, and excessive collagen deposition. PF is also an excessive repair response after tissue damage [93]. The pathological changes in ALI/ARDS are progressive. In the early stage of ALI/ARDS (within 7 days), the pathological changes in the lung tissue are dominated by exudation. After 7 days, ALI may develop into a fibrotic stage and generally develops into a fibrotic stage after 3 weeks [94]. Previous studies have shown that fibroblast proliferation and collagen secretion can be enhanced in the early stage of ALI. In other words, there are obvious fibrotic changes in the early stage of ALI [95, 96]. Alveolar epithelial cells (AECs) adhere tightly to adjacent cells or the basement membrane to protect the lungs from injury and infection. Under the action of pathogenic factors, the integrity of alveolar epithelial cells is disrupted, and the cells are rearranged, causing morphological and physiological changes in the cells. In addition, the phenotype of some epithelial cells is changed, and alveolar epithelial cells transform into mesenchymal cells. Alveolar epithelial cell-mesenchymal cell transdifferentiation is considered to be one of the important mechanisms of PF [97, 98].

Epithelial-mesenchymal transition (EMT) refers to the biological process in which epithelial cells are transformed into cells with a mesenchymal phenotype through specific procedures [99, 100]. EMT plays an important role in embryonic development, chronic inflammation, tissue reconstruction, cancer metastasis, and fibrotic diseases [100–102]. The main features of EMT are the decreased expression of cell adhesion molecules (such as E-cadherin), the conversion of the cytokeratin cytoskeleton into a vimentin-based cytoskeleton, and the acquisition of morphological characteristics of mesenchymal cells [103, 104]. During the EMT process, epithelial cells lose epithelial phenotypic characteristics, such as the ability to connect to the basement membrane, and acquire mesenchymal phenotypic characteristics, such as high migration and invasion capabilities, resistance to apoptosis, and the ability to degrade extracellular matrix [103, 104]. TGF-*β*/Smad is one of the main signaling pathways that promote EMT, and it is also a classic signal transduction pathway for the occurrence of pulmonary fibrosis [105]. In the TGF-*β*/Smad signaling pathway, Smad3 plays a particularly important role in the development of PF [105]. In addition, zinc finger E-box binding homeobox (ZEB) is the main regulator of TGF*β*-mediated signaling pathways. TGF*β* and its downstream signal factor Smad3 upregulate the expression of ZEBs, and signal transduction pathways such as p38/MAPKs can also induce or synergistically induce EMT transdifferentiation signal transduction in alveolar type II epithelial cells to cause the upregulation of ZEB protein expression [105, 106]. ZEB exerts an inhibitory effect on the expression of E-cadherin by binding with the promoter of E-cadherin, and then AT-II cells transdifferentiate from epithelial cells to mesenchymal cells [105, 106]. Zhou et al. [107] confirmed that alveolar epithelial cells could increase the level of reactive oxygen species (ROS) in mitochondria under hypoxia, and ROS could upregulate the expression of HIF-1*α* and TGF-*β*1 and promote the progression of EMT. Du et al. [108] confirmed that atorvastatin could attenuate paraquat-induced pulmonary fibrosis by downregulating the HIF-1*α*/*β*-catenin pathway and inhibiting EMT.

## 7. Strategies to Reduce Acute Lung Injury by Inhibiting HIF-1 (Table 1)

### 7.1. Low-Molecular-Weight Heparin (LMWH)

Heparin includes unfractionated heparin and LMWH [109]. LMWH is a class of low molecular weight heparin prepared by depolymerization of ordinary heparin. LMWH is a promising anticoagulant due to its excellent antithrombotic effect, high bioavailability, and low bleeding risk [109]. Due to its anti-inflammatory properties, LMWH can significantly reduce the permeability of microvessels, the migration of neutrophils, the expression of the lung plasminogen activator inhibitor 1 (PAI-1) gene, and the production of active PAI-1 and stabilize the dysregulated coagulation and fibrinolytic systems [110]. Previous studies have shown that LMWH prevents endotoxin-induced ALI and suppresses systemic inflammation [111, 112]. Severe inflammation leads to the activation of coagulation, and coagulation also affects the progression of inflammation. Accumulating evidence shows that inflammation and coagulation interact with each other [113–115]. Heparin has been shown to have both anticoagulant and anti-inflammatory effects [116]. Li et al. reported that LMWH can inhibit the expression of HIF-1*α*, VEGF, and TGF-*β*1 and have an anti-inflammatory effect. Li et al. [117] indicated that LMWH inhibits oxidative stress, inflammation, and apoptosis and leads to substantial improvements in pathological, physiological, and functional impairments by inhibiting HIF-1*α* signaling.

### 7.2. Emodin

*Rheum officinale*, a traditional Chinese medicine (TCM), has been used to treat pulmonary diseases in China for over a thousand years and can ameliorate inflammatory responses [118]. Emodin, one of the main active components of *Rheum officinale*, has a variety of biological activities, including anti-inflammatory, antioxidant, antitumor, and vasodilating activities [36, 119–121]. Emodin can reduce the expression of NF-*κ*B, thereby reducing the inflammatory response in ALI [122]. Mammalian target of rapamycin (mTOR) is involved in the regulation of the biological activities of pulmonary vascular endothelial cells and alveolar epithelial cells in ALI and therefore plays an important role in the occurrence and development of ALI [123, 124]. The activation of the mTOR signaling pathway may regulate the translocation of HIF-1*α* from the cytoplasm to the nucleus, thereby increasing the expression of VEGF, increasing the production of inflammatory factors, and exacerbating ALI [123, 125]. Li et al. [126] showed that emodin can improve the pathological changes in ALI induced by lipopolysaccharide through the mTOR/HIF-1*α*/VEGF signaling pathway and significantly reduce the expression of various inflammatory factors, including TNF-*α*, IL-1*β*, and IL-6.

### 7.3. Atorvastatin

Statins are a class of 3-hydroxy-3-methylglutaryl-coenzyme A (HMG-CoA) reductase inhibitors that are widely used in lipid-lowering drugs [127]. In recent years, in addition to the lipid-lowering effects of statins, their pleiotropic effects have also received increasing attention [128, 129]. Statins have been found to be beneficial for patients with chronic obstructive pulmonary disease, pneumonia, and pulmonary fibrosis [130–132]. Statins accelerated ubiquitin/proteasome-dependent degradation of HIF-1*α* [133]. Atorvastatin is a commonly used statin that has a significant protective effect against lung injury and PF [134–136]. Du et al. [108] found that atorvastatin could reduce ALI and PF caused by paraquat. Mechanistically, atorvastatin inhibits the EMT process by inhibiting the HIF-1*α*/*β*-catenin pathway and reducing PF after ALI.

### 7.4. Penehyclidine Hydrochloride (PHC)

PHC is a selective anticholinergic drug that acts on M receptor subtypes (M1, M2, and M3), and it is most selective for M1 and M3 receptors and less effective on the M2 receptor [137]. PHC can alleviate pulmonary capillary spasm, improve local microcirculation, reduce edema, and inhibit the production of inflammatory mediators [138, 139]. Previous studies have found that PHC could reduce renal ischemia/reperfusion-induced ALI, inhibit inflammation through the NF-*κ*B pathway, and improve the leakage of pulmonary blood vessels in lung tissue [140]. In addition, PHC has been suggested to ameliorate severe pancreatitis-related acute lung injury (PALI) by inhibiting the expression of inflammatory factors [141, 142]. Zhu et al. [143] showed that PHC could downregulate inflammatory factors such as NF-*κ*B and ILs by inhibiting HIF-1*α*, IL-1*β*, and IL-6 expression, thereby improving ALI.

### 7.5. Insulin and PEG-*b*-(PELG-*g*-PLL)

Insulin pretreatment can reduce ALI mediated by influenza virus infection [144]. However, the half-life of insulin is short, the dose is difficult to control, and frequent insulin injections increase the suffering of the patient. Tong et al. [145] used the block copolymer PEG-*b*-(PELG-*g*-PLL), which serves as a carrier, protects insulin from rapid degradation in the body, and can reinforce efficacy and reduce dosage and side effects. Insulin and PEG-*b*-(PELG-*g*-PLL) treatments are significantly protected against RI/R-induced ALI in rats. Insulin and insulin/PEG-*b*-(PELG-*g*-PLL) pretreatment can inhibit the activation of HIF-1*α* to reduce the activity of MPO and reduce the expression of VEGF by downregulating HIF-1*α* to decrease the inflammatory response in pulmonary tissues [145].

### 7.6. 3,5,4′-Tri-O-Acetylresveratrol

Resveratrol is a polyphenol extracted from plants especially Chinese traditional medicine such as knotweed, grapes, and nuts [146]. Resveratrol has a variety of pharmacological effects, such as antitumor, immunoregulatory, anti-inflammatory, antioxidant, and neuroprotective effects [147–151]. 3,5,4′-Tri-O-acetylresveratrol is the precursor of resveratrol, which overcomes the shortcomings of resveratrol, including weak pharmacokinetics, low bioavailability, and short-lived biological half-life [152]. Chiang et al. [127] showed that pretreatment with different doses of 3,5,4′-tri-O-acetylresveratrol improved seawater-induced lung histopathological changes, alleviated lung edema, reduced the production of inflammatory mediators, including TNF-*α* and IL-1*β*, inhibited malonaldehyde (MDA) activity, and enhanced total superoxide dismutase (T-SOD) activity, which was possibly associated with the inhibition of NF-*κ*B and HIF-1*α*. 3,5,4′-Tri-O-acetylresveratrol exhibited a protective effect on ALI by inhibiting oxidative stress and the inflammatory response, which may also involve the suppression of HIF-1*α* [127].

### 7.7. Tanshinone IIA

Danshen is a common herbal medicine that has been used clinically in China and many other Asian countries to prevent or control cardiovascular diseases [153]. Tanshinone IIA (TIIA), a derivative of phenanthrenequinone and one of the key components of Danshen, has been reported to have immunoregulatory, anti-inflammatory, and antioxidative properties [154, 155]. TIIA can alleviate lipopolysaccharide-induced ALI by inhibiting HIF-1*α* [156]. The possible mechanisms by which TIIA downregulates the expression of HIF-1*α* [156] are as follows: (1) TIIA cannot inhibit lipopolysaccharide-induced HIF-1*α* mRNA expression, but TIIA blocks the synthesis of HIF-1*α* protein by inhibiting the PI3K/AKT and MAPK pathways and related protein translation regulators such as p70S6K1, S6 ribosomal proteins, 4E-BP1, and eIF4E. (2) TIIA enhances the protein degradation of HIF-1*α* through the proteasome pathway.

### 7.8. COMP-Ang1

Angiopoietin-1 (Ang I) has potential therapeutic applications in inducing angiogenesis, enhancing endothelial cell survival, and stimulating and vascular stabilization [157]. Ang I also reduces the cytokine-induced expression of ICAM-1, vascular cell adhesion molecule 1 (VCAM-1), E-selectin, and tissue factors, thereby affecting the activation of endothelial cells and the adhesion and migration of leukocytes [158]. However, the aggregation and insolubility caused by the high-level structure of disulfide bonds hinder the production of Ang I. Cho et al. [159] developed a soluble, stable, and effective variant of Ang 1 (COMP-AngI). When lung tissue is exposed to oxidative stress, high levels of ROS can cause pathophysiological changes in lung tissue. ROS can induce the expression of VEGF to disrupt endothelial barrier function and subsequently increase the barrier permeability to fluids, macromolecules, and inflammatory cells [160, 161]. In a mouse model of acute lung injury caused by hydrogen peroxide (H2O2), COMP-Ang1 was shown to significantly reduce lung injury by inhibiting HIF-1*α* [162].

### 7.9. Propofol

Propofol (2,6-di-isopropylphenol) is a widely used intravenous anesthetic and sedative that is increasingly used to treat traumatic head injuries, epilepsy, delirium tremor, and asthma and to sedate critically ill patients [163]. Propofol has anti-inflammatory and antioxidant effects: it inhibits the migration, phagocytosis, and oxidation of macrophages [164]. In addition, propofol also inhibits the expression levels of a variety of inflammatory cytokines such as TNF-*α*, IL-*β*, and IL-6 [165]. Propofol can also reduce apoptosis and upregulate the expression of endothelial nitric oxide synthase (eNOS) in human endothelial cells [166]. Propofol could alleviate acute lung injury induced by endotoxin in a mouse model of sepsis [167, 168]. Yeh et al. [169] found that in a mouse model of endotoxin shock, a mild hypothermic dose of propofol could downregulate the expression of HIF-1*α* and inflammatory cytokines such as IL-6, IL-8, and TNF-*α* in lung epithelial cells, thereby reducing lung injury. Propofol reduced the expression of HIF-1*α* and its downstream proapoptotic gene BNIP3 in vitro and inhibited apoptosis in lung epithelial cells treated with lipopolysaccharide and desferrioxamine. Propofol can reduce acute lung injury by inhibiting HIF-1*α*.

## 8. Conclusion

The pathological process of ALI is very complicated and is characterized by excessive inflammation, alveolar epithelial cell apoptosis, and the destruction of the alveolar-capillary barrier, resulting in the accumulation of protein-rich pulmonary edema fluid. HIF-1 plays a major role in these processes. Exploring the role of HIF-1 in ALI and the pathogenesis of this common lung disease in a comprehensive and in-depth manner and finding ways to regulate HIF-1 will provide improved and more complete treatment for ALI.

## Figures and Tables

**Table 1:** The main treatments for ALI and their principal mechanisms.

Treatment agents	Principal mechanisms
LMWH	LMWH inhibits oxidative stress, inflammation, and apoptosis by inhibiting HIF-1*α* signaling.
Emodin	Emodin reduces inflammation through inhibiting the NF-*κ*B expression and mTOR/HIF-1*α*/VEGF signaling pathway.
Atorvastatin	Atorvastatin inhibits the EMT process by inhibiting the HIF-1*α*/*β*-catenin pathway and reducing PF after ALI.
PHC	PHC downregulates inflammatory factors by inhibiting HIF-1*α*, IL-1*β*, and IL-6 expression.
Insulin and PEG-*b*-(PELG-*g*-PLL)	Insulin and PEG-*b*-(PELG-*g*-PLL) inhibit the activation of HIF-1*α* to reduce the activity of MPO and reduce the expression of VEGF by downregulating HIF-1*α* to decrease the inflammatory response.
3,5,4′-Tri-O-acetylresveratrol	3,5,4′-tri-O-acetylresveratrol exhibited a protective effect on ALI by inhibiting oxidative stress and the inflammatory response, which may also involve the suppression of HIF-1*α*.
TIIA	(1) TIIA blocks the synthesis of HIF-1*α* protein by inhibiting the PI3K/AKT and MAPK pathways and related protein translation regulators such as p70S6K1, S6 ribosomal proteins, 4E-BP1, and eIF4E; (2) TIIA enhances the protein degradation of HIF-1*α* through the proteasome pathway.
COMP-Ang1	COMP-Ang1 reduces ALI by inhibiting HIF-1*α* and oxidative stress.
Propofol	Propofol reduces inflammatory cytokine expression and inhibits apoptosis by inhibiting HIF-1*α*.

## Data Availability

The data of this manuscript are available on request from the corresponding author.
